# Altered Spontaneous Brain Activity in Patients with Hemifacial Spasm: A Resting-State Functional MRI Study

**DOI:** 10.1371/journal.pone.0116849

**Published:** 2015-01-20

**Authors:** Ye Tu, Yongxu Wei, Kun Sun, Weiguo Zhao, Buwei Yu

**Affiliations:** 1 Department of Anesthesiology, Ruijin Hospital, Shanghai Jiaotong University School of Medicine, Shanghai, China; 2 Department of Neurosurgery, Ruijin Hospital, Shanghai Jiaotong University School of Medicine, Shanghai, China; 3 Department of Radiology, Ruijin Hospital, Shanghai Jiaotong University School of Medicine, Shanghai, China; Beijing Normal University, Beijing 100875, CHINA

## Abstract

Resting-state functional magnetic resonance imaging (fMRI) has been used to detect the alterations of spontaneous neuronal activity in various neurological and neuropsychiatric diseases, but rarely in hemifacial spasm (HFS), a nervous system disorder. We used resting-state fMRI with regional homogeneity (ReHo) analysis to investigate changes in spontaneous brain activity of patients with HFS and to determine the relationship of these functional changes with clinical features. Thirty patients with HFS and 33 age-, sex-, and education-matched healthy controls were included in this study. Compared with controls, HFS patients had significantly decreased ReHo values in left middle frontal gyrus (MFG), left medial cingulate cortex (MCC), left lingual gyrus, right superior temporal gyrus (STG) and right precuneus; and increased ReHo values in left precentral gyrus, anterior cingulate cortex (ACC), right brainstem, and right cerebellum. Furthermore, the mean ReHo value in brainstem showed a positive correlation with the spasm severity (r = 0.404, *p* = 0.027), and the mean ReHo value in MFG was inversely related with spasm severity in HFS group (r = -0.398, *p* = 0.028). This study reveals that HFS is associated with abnormal spontaneous brain activity in brain regions most involved in motor control and blinking movement. The disturbances of spontaneous brain activity reflected by ReHo measurements may provide insights into the neurological pathophysiology of HFS.

## Introduction

Hemifacial spasm (HFS) is characterized by unilateral, involuntary, paroxysmal contraction of the musculature innervated by the ipsilateral facial nerve [1]. Even though HFS is not a life threatening condition, it can lead to significant visual and verbal disability, social embarrassment and adversely affect quality of life [2]. However, the pathogenesis of HFS remains poorly understood. Neurovascular compression of the root exit zone (REZ) of the facial nerve is considered to be the most common causes of HFS [3]. An alternative hypothesis is hyperexcitability of the facial nucleus [4]. Emerging studies of HFS have already highlighted disease-related alterations in brain regions, extending the traditional focus on neurovascular conflict. Researchers who have performed positron emission tomography (PET) have detected bilateral cerebral glucose hypermetabolism in the thalamus of patients with HFS [5]. The transcranial magnetic stimulation (TMS) studies have shown that deficient intracortical inhibition plays a role in the pathophysioly of HFS [6]. Thus, these studies suggest that the presence of both peripheral and central mechanisms of patients with HFS.

FMRI is valuable tool in exploring the pathophysiology of neurological and neuropsychiatric diseases [7, 8]. In the past decade, with mounting evidence of task-related fMRI studies, whereas, which has been suggested and generally accepted, that alterations in cerebral function cannot only be observed during task performance, but also during the resting state. Therefore, the field has begun to focus on functional abnormalities during the resting state. Resting-state fMRI, a promising neuroimaging technique that can measure spontaneous neural activity has been widely used in investigating the neuropathphysiology of movement disorders including dystonia, Parkinson’s disease and tremor [9–11]. ReHo first proposed by Zang et al, a robust and reliable index, can effectively evaluate resting-state brain activity [12, 13]. ReHo is calculated using Kendall’s coefficient of concordance (KCC), which evaluates similarities between the time series of a given voxel and its nearest neighbors [14]. As such, ReHo reflects the local coherence of spontaneous neuronal activity [15].

Little is known about the changes in the local synchronization of spontaneous fMRI signals that occur in HFS patients during the resting state. We hypothesized that ReHo of resting-state brain activity would be different between patients with HFS and healthy controls, particularly in brain regions that have been implicated in facial motor control. The aims of the present study were to explore alterations of regional neural activity by using resting-state fMRI with ReHo method, and to assess the association between these alterations of intrinsic neural activity and clinical features in HFS patients.

## Methods and Materials

### Participants

The study was approved by the Ethics Committee of Ruijin Hospital, Shanghai Jiaotong University School of Medicine. The protocol adhered to the Declaration of Helsinki, and all participants’ written informed consents were obtained prior to taking part in the study. A total of 63 participants were recruited in this study: 30 left-sided HFS patients and 33 age-, sex- and education matched healthy controls from Neurosurgery Department, Ruijin Hospital, Shanghai Jiaotong University School of Medicine and local community. All subjects were right-handed according to the Edinburgh Inventory [16]. Diagnosis of HFS is based on clinical phenomenology (the spasms usually start as “twitching” of the lower eyelid, followed by involvement of the other periorbital, facial, perioral, and platysma muscles), and was determined by two experienced clinical neurosurgeons (WG Zhao, YX Wei with 27 and 5 years of experience in clinical neurosurgery, respectively). Known causes of secondary HFS were excluded on the basis of medical histories, neurological examination, laboratory investigation and conventional MRI. All patients have neither other neurological and neuropsychiatric abnormalities. None of the subjects were using neuropsychiatric drugs. The spam severity in all patients was accessed according to the Jankovic disability rating scale (0–normal, 1–slight disability, no functional impairment, 2–moderate disability, no functional impairment, 3–moderate disability, functional impairment and 4–incapacitated) [17]. Disease durations were calculated from symptom onset to scan date in years.

### Data Acquisition

All MR images were acquired using the GE Signa HDxt 3.0T scanner (General Electric Medical Systems, USA) with a standard 8-channel head coil. Resting-state fMRI data were acquired using an echo-planar image (EPI) pulse sequence with 33 axial slices, thickness/gap = 4.0/0 mm, matrix = 64 × 64, TR = 2000 ms, TE = 40 ms, flip angle = 90°, FOV = 240 × 240 mm. A total of 210 time points was obtained in 7 min. High-resolution three-dimensional T1 (TR = 5.8 ms, TE = 1.8 ms, flip angle = 12°, thickness/gap = 1.0/0 mm, 196 sagittal slices, FOV = 256 × 256 mm, matrix = 256 × 256) data were also acquired.

### Data Analysis

Preprocessing was performed using the SPM8 (http://www.fil.ion.ucl.ac.uk/spm) and Data Processing Assistant for Resting-State fMRI (DPARSF, http://rest.restfmri.net) [18]. The first 10 volumes were discarded to allow for scanner calibration and participants’ adaptation to the scanning environment. The remaining 200 volumes were analyzed. The steps included slice timing, head-motion correction, spatial normalization in Montreal Neurological Institute (MNI) space and resampling with 3 × 3 × 3 mm^3^ resolution. Participants with head motion > 2.0 mm of translation or > 2.0° of rotation in any direction were excluded from further analysis. As described in previous study, head motion can significantly influence measures and results derived from the fMRI scan [19]. Hence, we examined the group differences of head motion by using two-sample t-tests according to mean framewise displacement (FD) Jenkinson measurement [20, 21]. Resting-State fMRI Data Analysis Toolkit (REST) was then used for the following steps [22]: The linear trend of the fMRI data was removed, and band-pass filtering (0.01–0.08Hz) was conducted to decrease the impact of high-frequency physiological noise and the very low-frequency drift [23]. Individual ReHo map was generated by calculating the KCC of the time series of a given voxel with those of its neighbors (26 voxels) in a voxel-wise way [12, 24]. Afterwards a whole-brain mask (70831 voxels; made from the MNI template) was adopted to remove the nonbrain tissues. For standardization purposes, the individual ReHo maps were divided by their own global mean KCC within the whole-brain mask. Then spatial smoothing was performed on the standardized individual ReHo maps with a Gaussian kernel of 4 mm full-width at half maximum (FWHM) [25].

### Statistical Analysis

Demographic and clinical data were analyzed using the statistical package SPSS 17.0, differences of age, sex, and years of education between patients and health controls were analyzed using two-sample t-tests; comparison of gender was conducted using χ^2^ test. The threshold for all statistical significance was set at *p* < 0.05.

For ReHo, two-sample t-test with age, gender and mean FD as covariates between the HFS group and the control group were conducted in a whole-brain voxel-wise way by using REST toolbox. Voxels with *p* < 0.01 and cluster size > 486mm^3^ (18 voxels), which resulted in a corrected threshold of *p* < 0.05 determined by AlphaSim (rmm = 5 mm; http://afni.nih.gov/afni/docpdf/AlphaSim.pdf), were regarded to show a significant difference between the two groups. Brain regions showing significant differences (*p* < 0.05, corrected) in regional ReHo between groups were first created for regions of interest (ROI) masks. These ROI masks were then back-projected to the smoothed images of each patient, and mean ReHo values of the ROIs in each patient were also extracted using REST.

To further explore brain regions which may relate to clinical variables of HFS patients, correlation analysis between mean ReHo from each ROI and clinical features, (*a*) disease duration (years) and (*b*) spasm severity (Jankovic disability rating scale) were performed, with *p* < 0.05 (two-tailed) considered statistical significance.

## Results

### Demographic and clinical characteristics

Demographic and clinical data for all subjects were summarized in Table 1. There were no significant differences in age, gender and years of education between HFS patients and healthy controls.

**Table 1:** Demographic and clinical characteristics of participants in this study.

	**HFS patients**	**Healthy controls**	***p*-value**
Characteristics	n = 30	n = 33	
Age (year)	49.7 ± 8.0	50.9 ± 7.4	0.525^a^
Gender (male: female)	15: 15	11: 22	0.208^b^
Education (years)	9.7 ± 3.1	11.5 ± 4.0	0.522^a^
HFS duration (years)	6.8 ± 4.2	N/A	N/A
Spasm severity	2.8 ± 0.8	N/A	N/A

^a^The *p* value for difference between the two groups was obtained by two-sample t test.

^b^The *p* value for gender distribution was obtained by chi-square test. HFS: hemifacial spasm.

### FMRI Results

**Figure 1:**
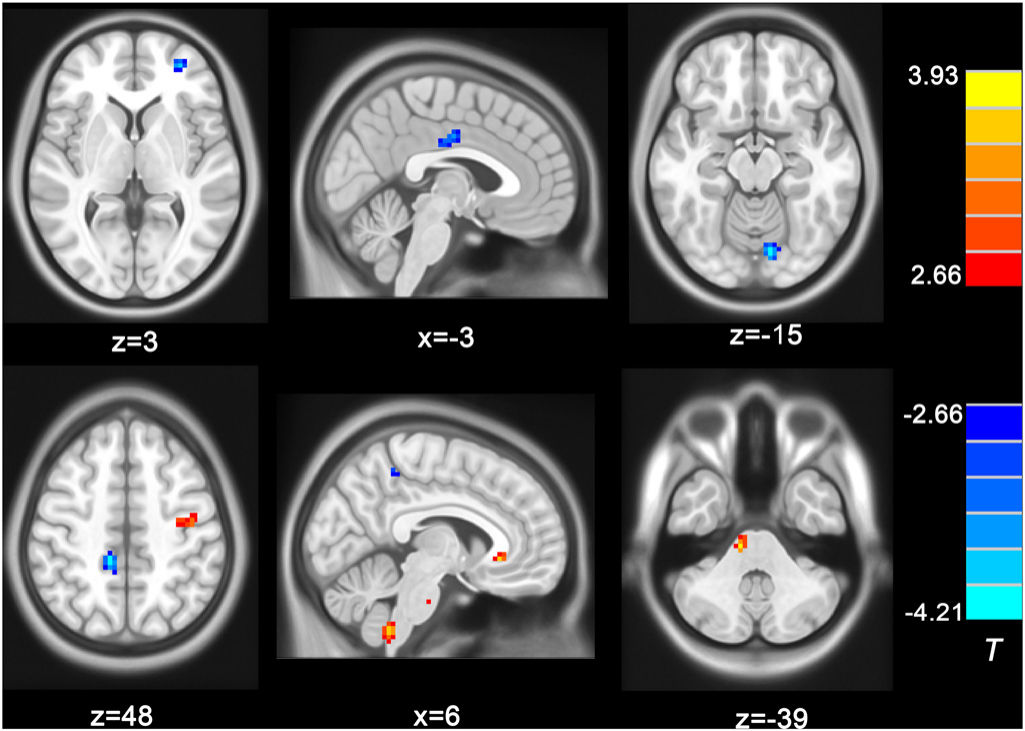
Clusters showing significant ReHo differences between two groups. The cold colors indicate lower ReHo in HFS group than healthy control group, while the warm colors mean vice versa (*p* < 0.05, AlphaSim corrected). Color bars represent the t value of the group analysis. Left in the figure indicates the right side of the brain.

ReHo changes in HFS patients as shown in Fig. 1 and Table 2. The left MFG, left MCC, left lingual gyrus, right STG and the right precuneus demonstrated a considerable decrease ReHo in the HFS patients compared with the healthy controls; while the left precentral gyrus, left ACC, right brainstem and cerebellum showed increased ReHo in the HFS patients compared with healthy controls.

**Table 2:** Clusters of significant ReHo differences between two groups.

**Regions**	**MNI coordinate**			**Peak t-score**	**Number of voxels**	**Volume (mm^3^)**
	**x**	**y**	**z**			
HFS > HCs						
Precentral gyrus (L)	-42	-9	57	3.38	38	1026
Brainstem (R)	12	-27	-39	3.93	26	702
Cerebellum (R)	6	-48	-57	3.55	27	675
Anterior cingulate cortex (R)	6	27	-6	3.53	20	540
HFS < HCs						
Middle frontal gyrus (L)	-30	51	3	-3.72	28	756
Superior temporal gyrus (R)	66	-51	15	-4.15	23	621
Medial cingulate cortex (L)	-3	-12	36	-3.67	30	810
Precuneus (R)	12	-39	48	-4.13	28	756
Lingual gyrus (L)	-9	-81	-15	-4.21	28	756

HFS: hemifacial spasm; HCs: healthy controls; MNI: Montreal Neurological Institute; L: left; R: right.

### Correlation between ReHo and Clinical Variables

**Figure 2:**
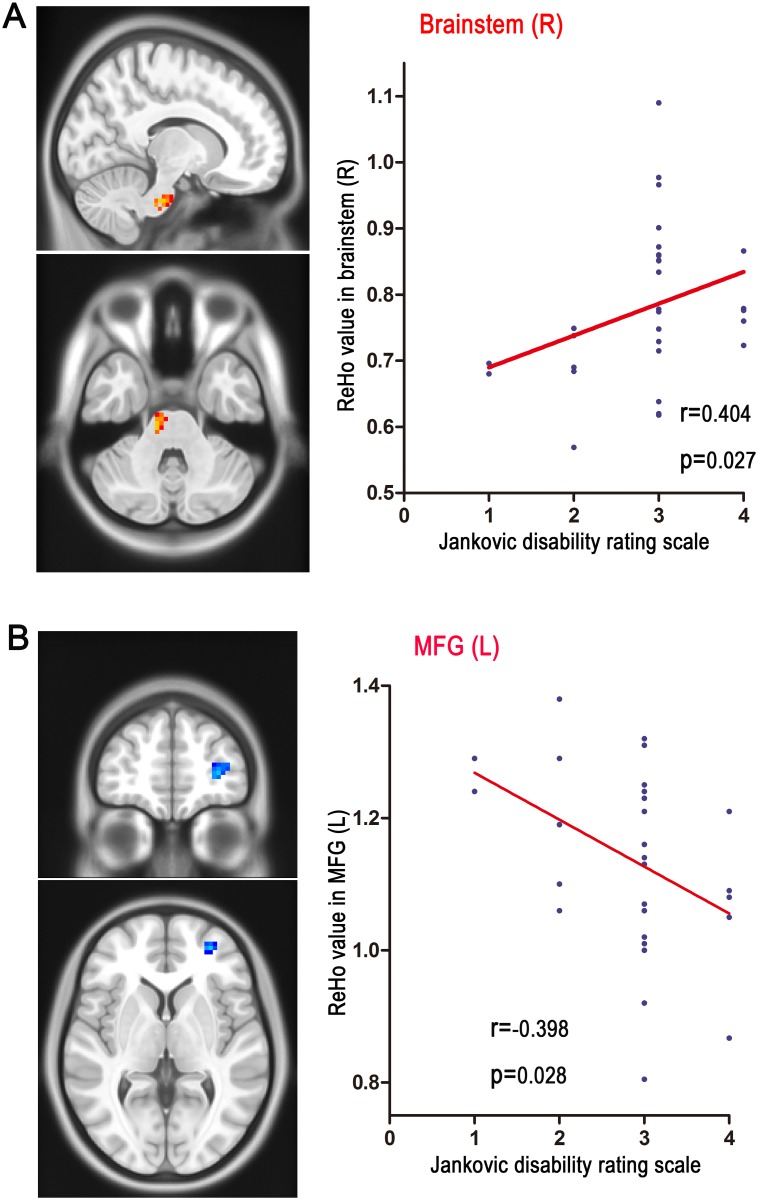
The correlation between spasm severity and the ReHo values in brainstem, as well as MFG in the HFS groups. **A.** the ReHo value in brainstem is positively correlates with spasm severity in the HFS group (r = 0.404, *p* = 0.027). **B.** the ReHo value in MFG is inversely correlates with spasm severity (r = -0.398, *p* = 0.028). HFS: hemifacial spasm MFG: middle frontal gyrus.

We examined the relationship between the disease duration, spasm severity and ReHo in regions with significant group differences. We found that the ReHo in the brainstem was positively related with the spasm severity in patients group (r = 0.404, *p* = 0.027 as shown in Fig. 2A); additionally, a negative correlation was detected between ReHo in the MFG and spasm severity in the HFS group (r = -0.398, *p* = 0.028), as shown in Fig. 2B). However, correlation analyses in the HFS group did not reveal any significant associations between the ReHo value in regions with significant group differences and disease duration.

## Discussion

To the best of our knowledge, this is the first resting-state fMRI study to examine spontaneous neuronal activity in HFS patients. Compared with healthy controls, HFS patients showed widely distributed ReHo alterations, such as increased ReHo in precentral gyrus, brainstem, cerebellum and ACC, and decreased ReHo in the MFG, MCC, STG, precuneus and lingual gyrus. Moreover, the ReHo in brainstem was positively correlated with spasm severity in the HFS group (r = 0.404, *p* = 0.027), and the ReHo in MFG was inversely correlated with spasm severity (r = -0.398, *p* = 0.028) of HFS patients. The present study provided the first evidence for alterations of distributed cerebral function at resting state in HFS patients.

The main findings of this study are the increased ReHo in the precentral gyrus [primary motor cortex (M1) is located on the precentral gyrus] and decreased ReHo in MCC of HFS patients. Recent studies reveal the existence of multiple facial representations, these comprise M1, MCC, and ventral lateral premotor cortex [26]. Additionally, all facial muscular groups seem to be bilaterally represented, with predominant representation of the contralateral side [27]. Thus, the possible explanation for facial spasm in HFS patients could be related to the functional alteration of facial motor system. Overall, our results suggest that both the precentral gyrus and MCC are pivotal cortical areas for the pathophysiology of HFS.

Our results demonstrated increased ReHo in the brainstem (facial nucleus is a collection of neurons in the brainstem), and we also found that the ReHo in the brainstem was positively related with the spasm severity in patients group. This result was consistent with the hyperexcitability of bilateral facial nucleus identified by electrophysiology [28–30]. Our data support the hypothesis that functional changes within facial nucleus is a central mechanism responsible for HFS.

We observed that abnormal local coherence in MFG of HFS patients, and the ReHo in MFG inversely correlated with spasm severity of patients. Previous fMRI studies found that MFG was involved in motor inhibition [31, 32]. The impaired motor inhibition has been also reported in a wide range of movement disorders [10, 33]. According to the above-mentioned findings, the decreased ReHo in MFG indicated that HFS patients could be abnormal in inhibitory motor control. This inference needs further confirmation in future.

Our results showed that abnormal spontaneous neural activities in cerebellum of HFS patients. There are two possible explanations for functional changes in cerebellum. First, the cerebellum receive input from multiple cortical areas, and have been traditionally been thought to modulate motor control, also are implicated in a range of movement disorders [34–36]. The altered cerebellar function, may be related to impaired cerebellar inhibition of motor cortex. Second, the cerebellum receives extensive somatosensory input via spinocerebellar pathways, and the cerebellum would be a sensory organ [37]. The functional changes in cerebellum could be due to increased sensory input derived from involuntary muscle contraction of eyelids and facial spam.

Additionally, we observed decreased ReHo in STG and ACC of HFS patients. FMRI studies have shown that ACC and STG are activated in healthy individuals during the blinking suppression, and indicated that ACC as well as STG are involved in eye blinking [38–40]. The early symptom of HFS patients to appear is usually an increased rate of involuntary blinking. We infered the functional abnormality in these areas was likely to relate to frequent eyeblink.

In the present study, we also found that decreased ReHo in the precuneus of HFS patients. The precuneus is a region previously shown to be part of the default mode network (DMN), and functional activity in the DMN is higher during rest and reduced during cognitive activity [41]. This suggested that there were distinct differences in the DMN in HFS patients compared with healthy controls.

The increased ReHo in lingual gyrus of HFS patients is difficult to interpret, it is a brain structure that is linked to processing vision, given the lack of direct involvement of this cortical region in motor control [42]. Most HFS patients characterized by eyelid spasm, frequent eyelid closure could interfere with vision. Whether the functional abnormalities detected by ReHo might be related, at least in part, to nonmotor (eg, visual) aspects of HFS needs further exploration.

However, this study is limited in the following aspects. First, our findings were at most preliminary given the fact that the sample sizes of both groups are relatively small. Confirmative studies with larger sample sizes were necessary. Second, to ensure the homogeneity of the sample, patients with only left-sided HFS were included in the study. The generalization of our findings in ReHo abnormalities to right-sided HFS patients is subject to further investigation.

In conclusion, this study demonstrates that spontaneous brain activity is profoundly altered in HFS patients. Complementing previous finding on neurovascular compression, we found HFS was associated with functional brain changes during resting state. Overall, ReHo analyses are powerful and easily applicable clinical tools to assess neurobiological changes in HFS and may provide valuable insights into functional disruptions in HFS.
